# Treatment of a chronic recurrent fistulized tibial osteomyelitis: administration of a novel antibiotic-loaded bone substitute combined with a pedicular muscle flap sealing

**DOI:** 10.1007/s00590-012-0956-5

**Published:** 2012-02-19

**Authors:** Massimo Franceschini, Adriano Di Matteo, Hans Bösebeck, Hubert Büchner, Sebastian Vogt

**Affiliations:** 1 Heraeus Medical GmbH, Philipp-Reis-Str. 8/13, 61273 Wehrheim, Germany; 2 Ospedale Gaetano Pini, Milan, P.zza A. Ferrari, 1, 20122 Milan, Italy; 3 Orthopaedics and Trauma University Hospital Luigi Sacco, Via G.B. Grassi, 74, 20157 Milan, Italy

**Keywords:** Chronic osteomyelitis, Tibia, Antibiotic-loaded bone substitute, Lower limb, Pedicular muscle flap sealing

## Abstract

Persistent osteomyelitis is a severe and challenging problem in bone surgery. We describe a surgical intervention in a young adult which combines a bone debridement process, a plastic muscle flap sealing and the administration of a novel bone substitution material with anti-infective properties. After 1 year, the patient showed no signs or symptoms of a reoccurrence of infection with full load capacity of the treated leg.

## Introduction

Even a radical debridement after a long-lasting osteomyelitis/osteitis is often not sufficient enough to eradicate an infection and save functional bone structures [1, 2]. In critical cases, there is a danger of fatal bone loss due to the release of osteolytic enzymes produced by bacteria relevant to bone infection like some strains of Staphylococcus aureus, for example, [3]. Although the routine use of antibiotic prophylaxis in orthopaedic surgery has been shown to be beneficial, the effectiveness of intravenous antibiotic administration is often complicated by insufficient local drug delivery. Hence, the development of a bone substitute that can also serve as a local drug delivery system may be beneficial in patients with such indications or patients who are at risk for developing a bone infection [4].

The below described final surgical approach is an example of a surgically justified option for a treatment which primarily met the requirement of the young patient not to have a more invasive procedure.

## Case report

A young man 32 years of age suffered from a chronic tibial osteomyelitis with a secreting fistula. The patient gave a history of trauma of an open fracture (1°/2°Gustilo-Anderson) after a motorbike accident 7 years back, for which he underwent seven previous surgeries. The first treatment was an osteosynthesis with external fixator which has been put in place for 9 months. As the fracture did not reveal any improvement in healing, it was removed, and the leg was protected with a cast supported by an administration of cephalosporins and glycopeptides i.v. followed by oral anti-infectives. After 6 months, the first evidence of osteomyelitis occurred after a CT scan, MRI, US and scintigraphy was evaluated from the area of interest. It was treated with a glycopeptide and a penicillin plus penicillinase inhibitor i.v.

Despite the above mentioned high suspicion of a septic non-union, an intramedullary nailing was performed. This treatment led to a consolidation of the fracture site, but other surgical procedures and antibiotic therapy were required for treatment of the chronic tibial osteomyelitis. Later a nail removal procedure was accompanied by an i.v. cephalosporin and oral amoxicillin + clavulanate administration. First bacteriological cultures revealed uncommon pathogens like Staphylococcus lugdunensis and Bacteroides caccae.

About 3 years after the accident, therapies with other oral antibiotic administrations of amoxicillin + clavulanate, quinolones, rifampin and hyperbaric O_2_ therapy, and a fistulizing chronic osteomyelitis with positive cultures from Enterococcus followed by strains of Corynebacterium did not seem to resolve.

Approximately 5 years after the patient’s trauma, a new surgery with a bone and soft tissue debridement and coverage with transpositional proximal pedicular flap was performed followed by oral beta-lactamase-stable third-generation cephalosporin therapy.

This last surgical procedure was a failure as cultures from wound dehiscence revealed a persistent infection sustained by Stenotrophomonas maltophilia and Staphylococcus aureus (MSSA) strains.

**Fig. 1:**
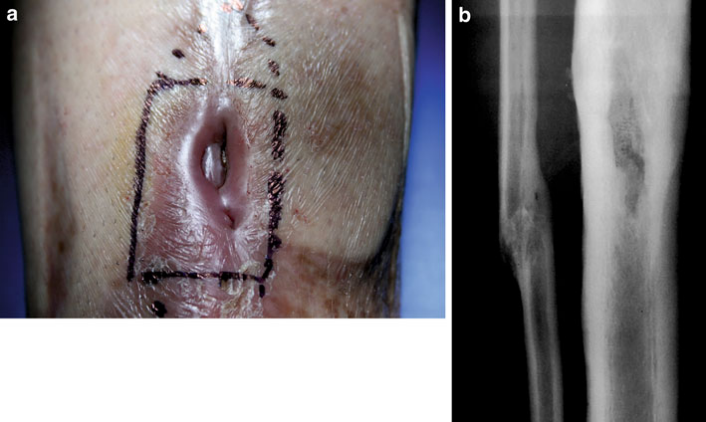
**a** The open fistula and the corresponding bone defect underneath **b** X-ray)

At this point, the patient came to our hospital with an open fistula and a tibial bone loss (Fig. 1a) expecting a treatment to end his clinical history. After clinical evaluation and radiological findings, a major procedure consisting in resection and bone transport with an Ilizarov Ex-fix was recommended [5, 6].

However, as the patients psychological profile did not permit this first surgical option, we were forced in trying a minor procedure consisting of a new radical debridement of the septic site, treatment of the bone loss with antibiotic-loaded bone substitutes (Herafill^®^ beads G, Heraeus Medical Wehrheim) and coverage of the skin with a new muscle flap [7, 8].

### Bone surgical management

The skin was marked over the fistula and excised with an oval-shaped incision. Preoperative examination of the skin defects showed that there was no need to minimize superficial debridement as we had planned a plastic reconstruction. No periosteum was present over the portion of bone exposed, which appeared just below the superficial tissue and the excised necrotic skin. Underneath a cortical defect (6.0 × 2.0 cm) was present with a cavity filled with an unidentified bone substitute.

All overlying and surrounding scars were excised until viable tissue was found [9]. Periostal stripping and devascularization were limited to what was absolutely required. Soft tissue and bone samples were collected and sent for cultural and histological examination. Some sequester consisting of bone substitutes not reabsorbed from previous surgery and pieces of necrotic cortex were removed, and the tibial canal was opened up both proximally and distally.

**Fig. 2:**
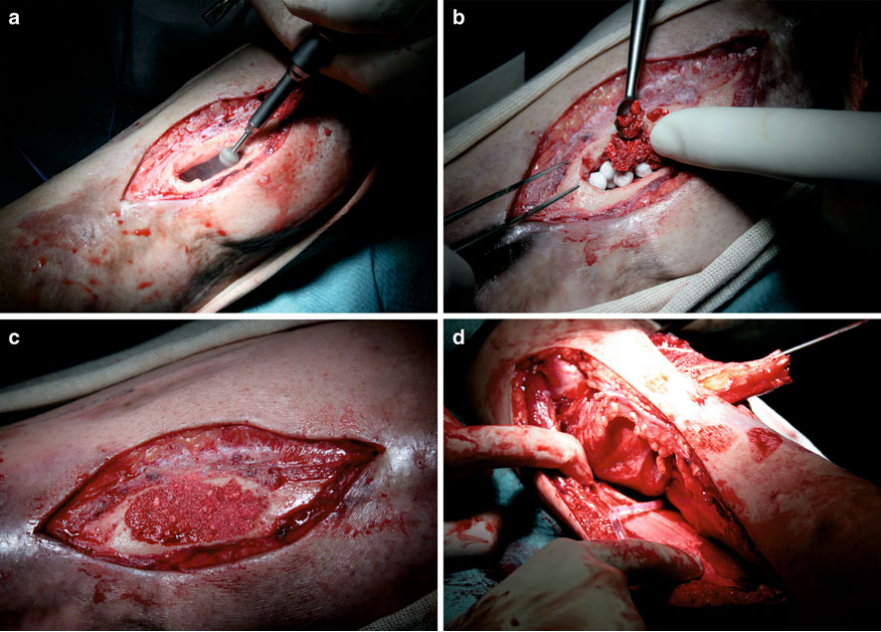
The surgical procedure from removing the fistularized scar tissue, radical debridement, application of Herafill^®^ beads G, sealing of the defect zone with a thin layer of a HA, the coverage with a vascularized muscle flap to final cutaneous wound closure

An accurate debridement of the endosteum was performed (Fig. 2a), edges of the bone void, which appeared avascularised, were reamed, up to where bleeding and vital tissue were present. However, it was possible to preserve two-thirds of the circumference of the tibia; therefore, there was no further action needed to avoid an onward weakening of the bone and create iatrogenic fractures [10].

Not less than five litres of normal saline was diluted with povidone-iodine (PVPI) to achieve a 0.35% povidone-iodine (3.5% Betadine) solution that was used as irrigation with pulse lavage [11]. Subsequent to the radical debridement, the bone defect treatment was followed by filling the medullary canal with 26 beads of Herafill^®^ beads G.

#### Material

Herafill^®^ beads G is a novel bioresorbable bone substitution material for bone void filling [12]. The 6-mm spherical beads are a compound of calcium sulphate, calciumcarbonate and tripalmitate as bonding additive. The 2.5 mg comprised amount of gentamicin is an adequate agent for infection processes. It is recommended for wounds with an extended open surface to decrease the contact zone of the Herafill^®^ beads G to soft tissues. The defect area in this case was covered with 5 mg of natural, highly purified, nanocrystalline carbonated hydroxyapatite of bovine origin (Fig. 2b, c).

### Plastic surgical management

After this procedure (debridement and partial tibial ostectomy and defect treatment), the wound, located between the medial and distal third of the leg, measured 10 × 5 cm. A flap of M. hemisoleus, based on a proximal pedicle, was employed. Due to its thickness and length, this part of muscle is suitable for distal wounds of the lower limb. Moreover, the harvesting of the muscle should have no impact on patient’s daily activities like standing or walking, because this function is well accounted for the contralateral M. hemisoleus and M. gastrocnemius, which together ensure proper flexion of the foot.

According to Mathes and Nahai’s classification [13], the muscle belongs to class II, supplied by the posterior tibial and peroneal arteries.

The patient lay in the supine position with the lower limb in extra-rotation. The incision was performed from a point between the medial M. malleolus and the Achilles tendon towards the proximal quarter of the shank, 1 cm posterior to the medial edge of the tibia.

After incision of the deep fascia, the layer was identified between M. soleus and M. gastrocnemius and the anterior borderline of M. soleus.

In a first approach, a dissection between M. gastrocnemius and the M. soleus was performed. Subsequently, M. soleus is separated from the deep posterior compartment, making sure not to incise the intermuscular fascia, in order to preserve the posterior tibial neurovascular fascicle. Distally, an accurate dissection between the muscle fibres and the Achilles tendon, which originates from the fusion of the deep fascia of M. gastrocnemii and the superficial fascia of M. soleus, was carried out.

On the lateral edge of the M. soleus, the dissection was continued to the tendon, sparing the main pedicle. The median line and the raphe, which divides M. soleus, were identified. Thus, the medial M. hemisoleus could be harvested and serve as a wound sealing. The flap was anchored to the acceptor site with sutures that comprised only the tendon and not the muscle (in order to avoid lacerations) (Fig. 2d).

The coverage with split-thickness skin grafts was delayed due to the high risk of infection. Negative pressure drainage was installed at the flap for 10 days post-operatively in order to secure the M. hemisoleus flap for the applied allograft underneath. After removal of the drainage, a granulation tissue on the superficial aspect of the muscle was observed. A split-thickness skin graft, obtained from the contralateral thigh, was positioned on the muscle flap, assisted by newly applied negative pressure drainage. All stitches were removed finally 10 days later.

## Results

The patient followed the desired proper healing course of a 12-month post-operative period. No signs of an ongoing or recurring infection were evident. CRP values remained in a normal range since the third post-operative week. Due to the plastic muscle surgery performed, the function of the shank was within the expected range and gave no limitations to the mobility of the patient. The patient maintained full mechanical stability after this final surgery.

**Fig. 3:**
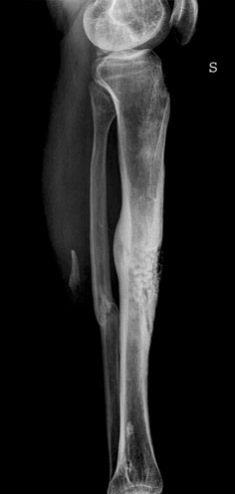
6 weeks post-op

**Fig. 4:**
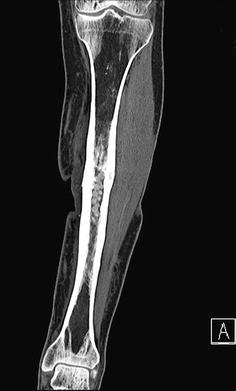
7 months post-op

**Fig. 5:**
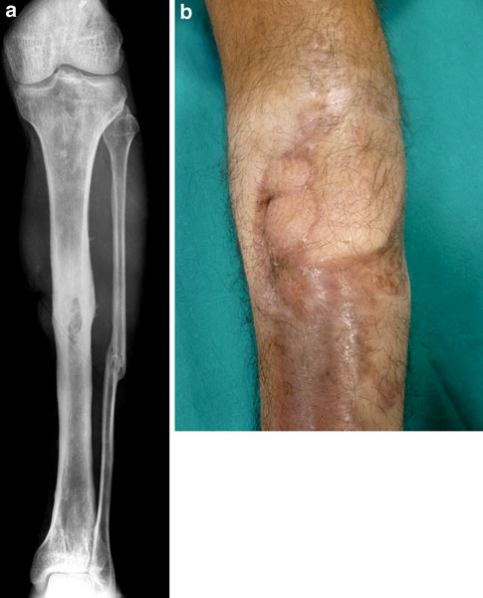
12 months post-op

The bone situation showed an undisturbed entire ossification of the tibia shown on native X-ray films (Fig. 3). Additional CT scans demonstrated that even tubular structures of the tibia (Fig. 4) could be preserved after the administration of the Herafill^®^ beads G. This bone substitution material was partly but not completely resorbed after 9 months. According to the definition of a GBR (guided bone regeneration) the neo-ossification followed the surface outline of the applied beads. No irritations between the bone void filling and the TCP sealing layer occurred, and the reconstruction remained stable (Fig. 5).

Due to the pedicular muscle flap surgery, a sufficient blood perfusion was ensured at the reconstruction site.

## Discussion

The primary indicated alternative of performing a distraction osteotomy using an Ilizarov could not be realized according to the mentioned psychological profile of the patient. Considering the actual status of the healing course, it can be stated that the surgical approach performing deep bone void debridement, administration of Herafill^®^ beads G and the process of sealing the wound by a vascularized pedicular muscle flap was a successful way of treatment that prevented the patient from a major surgery and an ongoing disturbance of the healing course. The chosen plastic surgical approach seemed to be a favourable way of wound sealing to ensure an undisturbed healing by an ongoing good perfusion [14].

Among a broad variety of bone substitution substances calcium sulphate/carbonate bone substitution beads proved to be a suitable osteoconductive material for bone reconstruction [15, 16]. This is in correlation with literature findings to use this material as an adequate local carrier system for antibiotic release [17, 18].

The antibiotic gentamicin seems to be an appropriate and active agent to support tissue healing burdened by infection processes. To be able to provide concise judgment of the post-operative change of the bone tissue, it is very important to assess the operated structures from different views and eventually different scanning methods. Otherwise, details can be missed out or tubular structures may only turn out as solid forms.

Despite a permissible incomplete skin appearance, which from the view of plastic surgery can be treated later on, the bony reconstruction revealed the expected clinical outcome. Hence, the chosen surgical approach as well as the applied antibiotic regimen seems to demonstrate a successful therapy that has now lasted more than 12 months [19].
